# Intraplaque Expression of C-Reactive Protein Predicts Cardiovascular Events in Patients with Severe Atherosclerotic Carotid Artery Stenosis

**DOI:** 10.1155/2016/9153673

**Published:** 2016-09-21

**Authors:** Aldo Bonaventura, François Mach, Aline Roth, Sébastien Lenglet, Fabienne Burger, Karim J. Brandt, Aldo Pende, Maria Bertolotto, Giovanni Spinella, Bianca Pane, Domenico Palombo, Franco Dallegri, Michele Cea, Nicolas Vuilleumier, Fabrizio Montecucco, Federico Carbone

**Affiliations:** ^1^First Clinic of Internal Medicine, Department of Internal Medicine, University of Genoa, 6 Viale Benedetto XV, 16132 Genoa, Italy; ^2^Division of Cardiology, Foundation for Medical Researches, Department of Medical Specialties, University of Geneva, 64 Avenue de la Roseraie, 1211 Geneva, Switzerland; ^3^Unit of Toxicology, University Centre of Legal Medicine, Geneva-Lausanne, Rue Michel-Servet 1, 1211 Geneva, Switzerland; ^4^IRCCS AOU San Martino-IST, 10 Largo Benzi, 16132 Genoa, Italy; ^5^Vascular and Endovascular Surgery Unit, Department of Surgery, IRCCS AOU San Martino-IST, 10 Largo Benzi, 16132 Genoa, Italy; ^6^Clinic of Hematology, Department of Internal Medicine, University of Genoa, IRCCS AOU San Martino-IST, 10 Largo Benzi, 16132 Genoa, Italy; ^7^Division of Laboratory Medicine, Department of Genetics and Laboratory Medicine, Geneva University Hospitals, 4 Rue Gabrielle-Perret-Gentil, 1205 Geneva, Switzerland; ^8^Centre of Excellence for Biomedical Research (CEBR), University of Genoa, 9 Viale Benedetto XV, 16132 Genoa, Italy

## Abstract

Serum c-reactive protein (CRP) was suggested for the assessment of intermediate cardiovascular (CV) risk. Here, systemic or intraplaque CRP levels were investigated as predictors of major adverse cardiovascular events (MACEs) in patients with severe carotid stenosis. CRP levels were assessed in the serum and within different portions (upstream and downstream) of carotid plaques of 217 patients undergoing endarterectomy. The association between CRP and intraplaque lipids, collagen, neutrophils, smooth muscle cells (SMC), and macrophage subsets was determined. No correlation between serum CRP and intraplaque biomarkers was observed. In upstream portions, CRP content was directly correlated with intraplaque neutrophils, total macrophages, and M1 macrophages and inversely correlated with SMC content. In downstream portions, intraplaque CRP correlated with M1 and M2 macrophages. According to the cut-off point (CRP > 2.9%) identified by ROC analysis in upstream portions, Kaplan-Meier analysis showed that patients with high CRP levels had a greater rate of MACEs. This risk of MACEs increased independently of age, male gender, serum CRP, and statin use. In conclusion, in patients with severe carotid artery stenosis, high CRP levels within upstream portions of carotid plaques directly and positively correlate with intraplaque inflammatory cells and predict MACEs at an 18-month follow-up period.

## 1. Introduction

It is widely established that both adaptive and innate immunity tightly regulate atherogenesis [1]. Several soluble and intraplaque inflammatory mediators have been shown to variably influence immune and vascular cell functions. One of the most studied inflammatory molecules is c-reactive protein (CRP), a short pentraxin produced by the liver in response to interleukin- (IL-) 6 following a microbial trigger or tissue damage [2]. In human beings, CRP is considered as an acute phase protein, with serum levels ranging from less than 1.0 mg/L at baseline to up to 1000-fold higher levels during acute response [2]. CRP can bind to oxidized or degraded low-density lipoprotein (LDL) activating complement; it can induce the expression of adhesion molecules, mediate the uptake of LDL-cholesterol by macrophages, stimulate monocyte recruitment within the arterial wall, and increase the production of chemokines, such as monocyte chemoattractant protein-1 [3–5]. Over the past two decades, CRP has been tested for clinical use as a sensitive, nonspecific systemic marker of infection, inflammation, and tissue damage. In particular, it has been highlighted as a promising marker of coronary heart disease, ischemic stroke and cognitive impairment, vascular mortality, and death from different cancers [6, 7]. However, the impact of CRP as a predictor of cardiovascular (CV) events has been weakened by Danesh and colleagues [8]. Given the strong association that exists between chronic inflammatory diseases and the increased risk of coronary artery diseases, the question has been raised as to whether CRP is an innocent bystander of or an active player in proatherosclerotic mechanisms [9].

Carotid artery stenosis represents an independent risk factor for ischemic cerebrovascular disease and a direct cause of cognitive impairment, with an estimated prevalence of 9.3% for patients older than 70 years [10, 11]. In the Caucasian population, carotid atherosclerosis is usually found at the carotid artery bifurcation, involving the distal common carotid and the proximal internal carotid arteries. Systemic and intraplaque inflammation have been suggested to be potentially associated with plaque vulnerability [12]. Serum high sensitivity- (hs-) CRP has already been demonstrated as being directly correlated with carotid plaque vulnerability [13–15]. However, intraplaque CRP has been poorly investigated in human carotid plaques [15]. In coronary arteries, smooth muscle cells (SMC) have been found to release CRP in response to inflammatory cytokines [16, 17], underscoring a potential proatherosclerotic role of CRP in plaque rupture and thrombosis [18].

In the present study, we investigated the potential correlations between serum and intraplaque CRP levels and intraplaque inflammatory and vascular cells in patients with severe carotid artery stenosis. Furthermore, considering plaque heterogeneity, we assessed the potential prognostic value that CRP values of upstream and downstream portions of carotid plaques could have on major adverse cardiovascular events (MACEs) at 18-month follow-up.

## 2. Methods

### 2.1. Patients and Clinical Assessment

From March 2008 to June 2011, we enrolled 269 patients with extra cranial, high-grade stenosis (>70% luminal narrowing) of the internal carotid artery in an observational cohort at a single center (IRCCS Azienda Ospedaliera Universitaria San Martino-IST Istituto Nazionale per la Ricerca sul Cancro, Genoa, Italy). Some of the samples have already been used for analysis and published [19–21]. Among the total cohort (*n* = 269), 52 carotid endarterectomies were missing, leaving 217 samples available for analysis in the present substudy. As previously described [22], all patients had undergone carotid endarterectomy (CEA) according to the recommendations published by the North American Symptomatic Carotid Endarterectomy Trial [23], the European Carotid Surgery Trial [24], and the Asymptomatic Carotid Surgery trial [25]. The Ethics Committee of IRCCS Azienda Ospedaliera Universitaria San Martino-IST Istituto Nazionale per la Ricerca sul Cancro in Genoa, Italy, approved this protocol, performed in accordance with the guidelines of the Declaration of Helsinki. Patients gave informed consent before entering the study. On the day prior to CEA, serum samples were obtained to evaluate circulating markers of cardiovascular vulnerability. Medications are reported in Table 1 and were not modified in the 2 months prior to enrolment. Exclusion criteria were as follows: spontaneous cerebral embolism up to 30 minutes preoperatively or during the dissection phase of the operation, malignant hypertension, acute coronary artery diseases, any cardiac arrhythmias, congestive heart failure (II, III, and IV New York Heart Association classes), liver or renal disorder or function abnormalities, acute and chronic infectious diseases, autoimmune and rheumatic diseases, cancer, endocrine diseases, inflammatory bowel diseases and anti-inflammatory (other than aspirin) medications, oral anticoagulant treatments (other than heparin), and hormone, cytokine, or growth factor therapies.

### 2.2. Study Endpoints and Power Calculation

The primary endpoint of the study was to determine whether intraplaque levels of CRP at the time of CEA could predict the occurrence of MACEs, defined as fatal or nonfatal acute coronary syndrome and stroke, over an 18-month follow-up period. The secondary endpoint consisted in determining potential correlations between CRP and inflammation parameters, both in serum and within carotid plaques. Two independent investigators who were blinded to the biochemical and histological analyses adjudicated the study endpoints. Information was obtained during a check-up visit at 18 months following CEA and further confirmed by checking patients' medical files. All the endpoints were calculated based on data available for carotid plaques of 217 patients.

The sample size was computed based on a previous study showing a local difference of CRP levels in patients with stable and vulnerable carotid atherosclerotic plaques [15]. According to our power calculation for logrank test, the minimal sample size requested to detect a fourfold increase in the risk of a MACE [21] with a power of 80% and a two-sided alpha error of 5% was of 138 patients.

### 2.3. Detection of Biochemical and Inflammatory Biomarkers

Routine autoanalyzers were used to assay hematological parameters and blood chemistry, including total cholesterol, low- and high-density lipoprotein cholesterol, triglycerides, fibrinogen, and glycaemia. Serum levels CRP and interleukin- (IL-) 6 were measured by colorimetric enzyme-linked immunosorbent assay (ELISA) following the manufacturer's instructions (R&D Systems, Minneapolis, MN). The limits of detection were 31.25 pg/mL for CRP and 0.156 pg/mL for IL-6. Mean intra- and interassay coefficients of variation were <8% for all markers measured by ELISA.

### 2.4. Carotid Plaque Processing

Within few minutes after surgical excision, the internal carotid plaque specimens were transferred at 4°C to the laboratory for processing [20, 22]. All atherosclerotic plaques were cut perpendicular to the long axis through the point of maximum stenosis to obtain both an upstream and a downstream portion. Each portion was further divided perpendicular to the long axis in the middle in two subsegments. Half was snap-frozen in liquid nitrogen and stored at −80°C (mRNA isolation), and the other half was frozen in cryoembedding medium (OCT) for histological analyses.

### 2.5. Oil Red O and Sirius Red Staining on Carotid Plaques

Each portion frozen for histology was then divided into 8 sections, cut at an interval of 105 *μ*m from each other, and stained with oil red O or sirius red, as previously described [20, 22]. The sections were photographed under light microscopy in order to evaluate total collagen content and under polarization microscopy. Interstitial collagen subtypes were evaluated using polarized light illumination; under this condition thicker type I collagen fibers appeared orange or red, whereas thinner type III collagen fibers were yellow or green. Total, type I, and type III collagen content were quantified by MetaMorph*™* 6 software. Results were presented as percentages of stained area on total lesion area.

### 2.6. Immmunostaining of Endarterectomy Specimens

For each portion, the eight sections cut at 105 *μ*m intervals from each other were fixed in acetone at room temperature and immunostained with specific antibodies. Primary antibodies, such as anti-human smooth muscle actin (smooth muscle cells (SMC); dilution: 1 : 100; Dako Corporation, Glostrup, Denmark), anti-human CD66b (neutrophils; dilution: 1 : 50; Beckman Coulter, Nyon, Switzerland), and anti-human CRP (dilution: 1/100; Sigma-Aldrich, Saint Louis, MI), were used. For detecting macrophages, we used anti-human CD68 (marker of total macrophages; dilution: 1 : 100; Dako Corporation, CA), anti-human CD86 (marker of M1 macrophages; dilution: 1 : 100; GeneTex Inc., Irvine, CA), anti-human HLA-DR (marker of M1 macrophages; dilution: 1 : 100; Dako Corporation), and anti-human CD163 (marker of M2 macrophages; dilution: 1 : 50; AbD Serotec, Oxford, UK) [26]. Quantifications were performed using MetaMorph 6 software. Data were presented as percentages of stained area on total lesion area.

### 2.7. Real-Time Reverse Transcription-Polymerase Chain Reaction (RT-PCR)

Total mRNA was isolated with Tri-Reagent (MRC Inc.) from the upstream and downstream portions of carotid artery plaques. Reverse transcription was performed using the ImProm-II Reverse Transcription System (Promega, Madison, WI, USA) according to the manufacturer's instructions. RT-PCR (StepOne Plus, Applied Biosystems, Foster City, CA, USA) was carried out with the ABsoluteTM QPCR Mix (ABgene, Epsom, UK). Specific primers and probes were used to determine the mRNA expression of CRP and RPS13 (housekeeping gene) [27]: CRP forward, 5′-GCTTTTGGCCAGACAGACA-3′; CRP reverse, 5′-CGGTGCTTTGAGGGATACA-3′; CRP Taqman probe, 5′-CGAGGAAGGCTTTTGTGTTTCCC-3′; RPS13 forward, 5′-CGTCCCCACTTGGTTGAAG-3′; RPS13 reverse, 5′-CCGATCTGTGAAGGAGTAAGG-3′; and RPS13 Taqman probe, 5′-FAM-ACATCTGACGACGTGAAGGAGCAGATT-BHQ1-3′. The fold change of mRNA levels was calculated by the comparative C_t_ method. The measured C_t_ values were first normalised to the RPS13 internal control, by calculating delta C_t_ (ΔC_t_). This was reached by subtracting the RPS13 C_t_ values from the gene of interest C_t_ value. A delta delta C_t_ (ΔΔC_t_) was calculated by subtracting the designated baseline control group ΔC_t_ value from the study group ΔC_t_ values. The ΔΔC_t_ was then projected as a relative fold change with the following formula: 2-ΔΔC_t_.

### 2.8. Statistical Analysis

Analyses were performed with IBM SPSS Statistics for Windows, version 23.0 (IBM Co., Armonk, NY), and MedCalc 12.5 (MedCalc Software, Ostend, Belgium). In the clinical study, categorical data are presented as relative and absolute frequencies, whereas continuous variables are expressed as median and interquartile range (IQR). Intergroup comparisons were drawn by Fisher's exact test and Mann–Whitney *U* test, as appropriate. Ranked Spearman correlation coefficients were performed to establish correlations between serum and intraplaque biomarkers in both upstream and downstream portions of carotid plaques. The prognostic ability of CRP was evaluated on the basis of a receiver operator characteristic (ROC) curve, using MedCalc 12.5 (MedCalc Software, Ostend, Belgium). The area under the curve (AUC) was given with 95% confidence interval (CI) and the cut-off point of CRP was calculated maximizing the sensitivity in accordance with Youden's index. Kaplan-Meier survival analysis with logrank test was performed to estimate the cumulative event rate during 18 months after CEA and to calculate the corresponding risk difference according to CRP. Finally, the effect of CRP expression on MACE risk was estimated by Cox proportional hazards models and expressed with hazard ratios (HR) and 95% CI. In the multivariate model, we adjusted for age, male gender, serum hs-CRP level, and statin use. For all statistical analyses a two-sided *p* value <0.05 was considered as statistically significant.

## 3. Results

### 3.1. Patient Characteristics

Clinical and laboratory characteristics of the cohort are listed in Tables 1 and 2, respectively. Patient median age was 72 years, with a higher prevalence of males (62.7%) and hypertensive and dyslipidemic patients (72.4% and 57.6%, resp.) (Table 1). The median value of total white blood cells (WBC) was 7.10 × 10^9^/L, while the median value of serum hs-CRP was 2.38 mg/mL, suggesting low-grade systemic inflammation (Table 2).

### 3.2. Intraplaque but Not Systemic CRP Levels Correlate with Parameters of Inflammation within Atherosclerotic Plaques

**Figure 1:**
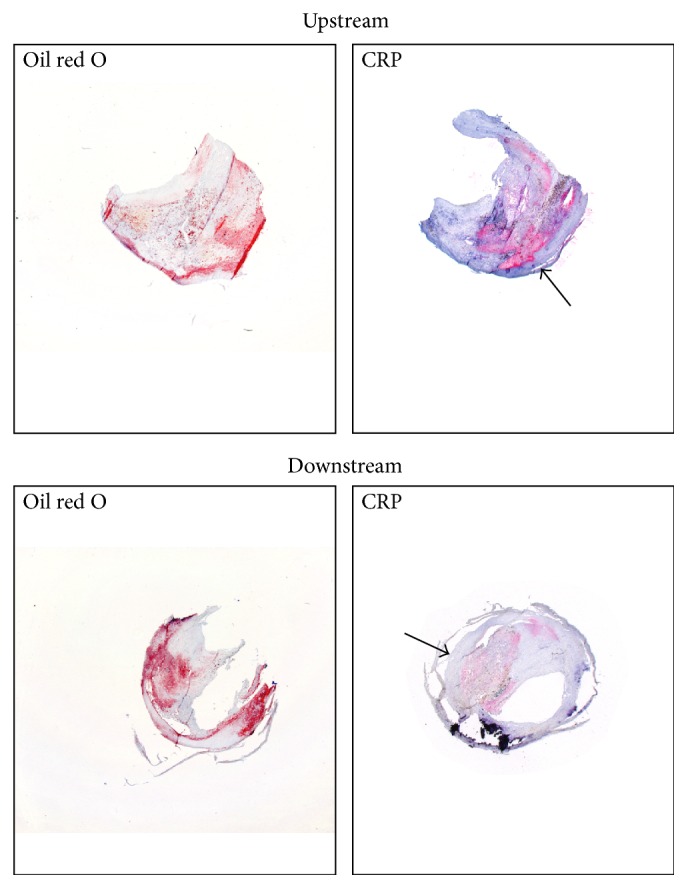
Representative microphotographs of human carotid atherosclerotic plaques. Immunostainings for total lipid content (oil red O) and CRP in upstream and downstream portions of human carotid artery plaques were performed. The arrows show CRP-positive areas.

Downstream portions of carotid plaques were characterized by increased neutrophil and M1 macrophage (CD86+ cells) content when compared to upstream regions (Table 3). Upstream portions of carotid plaques had significantly higher collagen (total, type I, and type III), smooth muscle cells (SMC), and M2 macrophage subsets when compared to downstream portions (Table 3). CRP expression in carotid samples at both the mRNA and protein level showed that intraplaque CRP content was well detectable at protein level in all samples of upstream and downstream portions of carotid plaques (Figure 1). The weak increase in intraplaque CRP levels noted in upstream as compared with downstream portions was nonsignificant (*p* = 0.070) (Table 3; Figure 1). Conversely, intraplaque CRP mRNA was almost undetectable (upstream plaques: detectable (i.e., <40 cycles at RT-PCR) in 29 of total 217 patients; downstream plaques: detectable in 29 of total 217 patients; data not shown).

In upstream portions, intraplaque CRP was directly correlated with neutrophils (*r* = 0.208; *p* = 0.008), total macrophages (*r* = 0.158; *p* = 0.046), and M1 subset (HLA-DR+ cells, *r* = 0.161; *p* = 0.042), whereas it was inversely correlated with SMC (*r* = −0.315; *p* < 0.001) (Table 4). In downstream portions, intraplaque CRP directly correlated with M1 (CD86+, *r* = 0.193; *p* = 0.014; HLA-DR+ cells, *r* = 0.226; *p* = 0.004) and M2 macrophage subsets (CD163+, *r* = 0.344, *p* < 0.001) (Table 4). No correlation was found between serum hs-CRP and intraplaque parameters of vulnerability, within both upstream and downstream portions of carotid plaques (Table 5).

### 3.3. CRP Levels in the Downstream Portion of Carotid Plaques Predict Future MACEs

**Figure 2:**
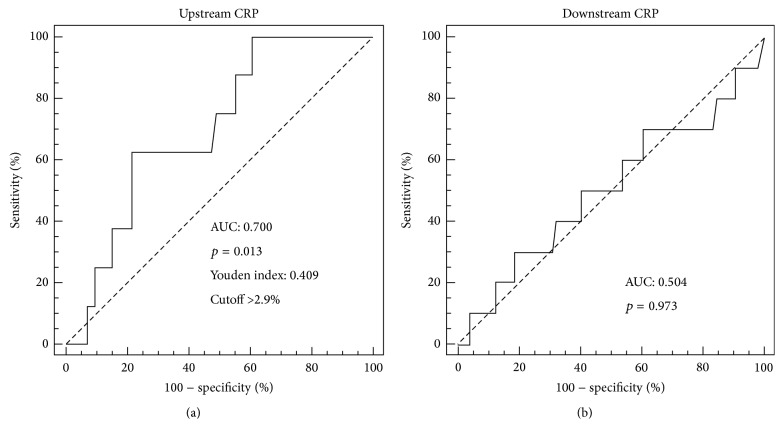
Receiver operator characteristic (ROC) curve analysis for CRP expression in upstream and downstream portions of carotid plaques. The predictive value of CRP expression toward the occurrence of major adverse cardiovascular events (MACEs) at 18-month follow-up was tested. (a) CRP expression in the upstream portion. (b) CRP expression in the downstream portion.

**Figure 3:**
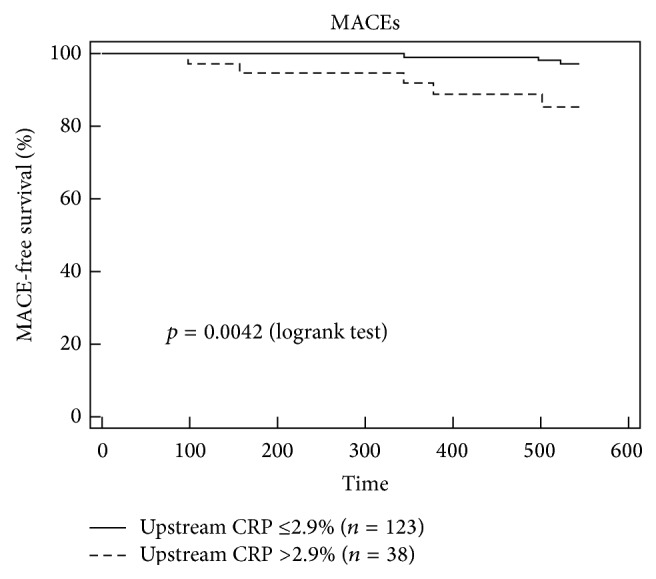
High upstream expression of CRP is associated with an increased rate of major adverse cardiovascular events (MACEs) at 18-month follow-up. Kaplan-Meier curve according to high (CRP: >2.9%) and low (CRP: ≤2.9%) CRP expression levels in upstream regions.

ROC curve analysis showed that CRP content in upstream (Figure 2(a)) but not in downstream (Figure 2(b)) portions of carotid plaques had significant prognostic accuracy to predict MACEs (AUC 0.700 [95% CI 0.623–0.770]; *p* = 0.013). In accordance with the Youden index, an upstream intraplaque CRP content of 2.9% was identified as the best cut-off point, having a sensitivity of 62.5% and a specificity of 78.4% (Figure 2(a)). After dividing upstream plaques into low (≤2.9%) and high (>2.9%) CRP groups, Kaplan-Meier analysis indicated that patients with high intraplaque CRP had a greater rate of MACEs than patients with low CRP levels (Figure 3). Of the 8 MACEs observed during the 18-month follow-up period, 5 occurred in the group with high CRP levels and 3 in the low CRP group. Cox proportional hazard regression analysis confirmed the abovementioned results. The risk of MACEs increased with increased CRP intraplaque content in the upstream portions of plaques (HR 6.22 [95% CI 1.49–26.05]; *p* = 0.012) (Table 6). These results remained statistically significant also after adjustment for age, male gender, hs-CRP, and statin use (HR 8.57 [95% CI 1.89–38.77]; *p* = 0.005) (Table 6).

## 4. Discussion

This study shows that intraplaque but not systemic CRP levels are associated with different inflammatory parameters of plaque vulnerability, particularly with regard to the upstream portions of carotid plaques. These findings might apparently be in contrast with previous studies demonstrating that serum CRP levels were positively associated with plaque vulnerability parameters [28–30]. On the other hand, CRP was already demonstrated to localize within early atherosclerotic plaques in human coronary arteries [31]. The proatherosclerotic role of “local” CRP deposits was presumed to actively contribute to intraplaque inflammation by recruiting monocyte/macrophages, promoting foam cell formation and inducing complement activation [32–35]. Devaraj and Jialal demonstrated a pivotal activity of CRP in polarizing human monocyte differentiation toward a proinflammatory M1 macrophage phenotype (via NF*κ*b and by binding to the Fc-*γ* receptors CD32 and CD64) and inhibiting M2 macrophages [36]. The pathophysiological relevance of intraplaque CRP was further supported by its positive association with clinical instability of CAD patients [18, 37]. Considering the heterogeneity of atherosclerotic plaques, the main novelty of our study was the demonstration that intraplaque CRP levels can differentially be associated with plaque vulnerability parameters, depending on where the plaque is positioned. In particular, CRP levels within plaque portions located upstream of the stenosis were positively correlated with vulnerability parameters (i.e., neutrophils and proinflammatory M1 macrophages) and negatively correlated with protective mediators (such as SMC). Differently from Wilson and coworkers [38], we were not able to show the positive association between intraplaque CRP protein and mRNA levels. In fact, CRP was detectable in only a few samples in the absence of relevant mRNA degradation. These results are in partial contrast with the assumption that intraplaque cells (vascular or inflammatory) might be able to express and release CRP [39]. Considering that a modest intraplaque amount of CRP was detectable in almost all carotid plaque samples, we might hypothesize that CRP infiltrates the subintimal region via the bloodstream [40]. We already speculated in 2008 about vascular CRP (localized within atherosclerotic plaques) as a promising CV risk factor that might play an active proatherosclerotic “intraplaque” role. These properties of intraplaque CRP might represent an interesting issue potentially explaining the reason why CRP serum levels were not related with the cardiovascular risk in several clinical articles [9]. To date, no clear evidence of the molecular and cellular mechanisms underlying intraplaque CRP infiltration versus production has been produced. The marginal role of CRP in mouse models of atherosclerosis did not help to improve knowledge. Taking into the account these limitations, and also confirming previous results [41, 42], our observational study only supports CRP intraplaque content as a proinflammatory mediator in human atherogenesis, without clarifying the mechanistic insights.

On the other hand, we were unable to find any association between serum concentrations of CRP and any intraplaque parameter of vulnerability. hs-CRP levels have previously been shown to be associated with atherothrombotic coronary plaques [43]. In addition, results from clinical trials have suggested serum hs-CRP as being a reliable parameter to monitor the efficacy of statin therapy in patients suffering from ST-elevation myocardial infarction [44]. Finally, some positive association between carotid plaque inflammation/vulnerability and circulating inflammatory biomarkers (i.e., CRP, tumor necrosis factor-*α*, and IL-6) has already been described [45, 46]. In contrast with the latter studies, our findings on systemic CRP were surprising in that they corroborate the findings by Grufman and colleagues that showed no significant association between plasma hs-CRP and intraplaque cytokine levels [47].

Another relevant finding of our study is the CV predictive value of CRP levels in upstream portions of carotid plaques. CRP content greater than 2.9% was correlated with a sixfold increase in the probability of MACEs, in both univariate and multivariate analyses. Moreover, upstream CRP content predicted MACEs independently of age, male gender, hs-CRP, and statin use. At the best of our knowledge, this is the first time that intraplaque but not circulating CRP levels were strongly correlated with MACEs in a cohort of patients with severe carotid stenosis.

This study has some limitations. First of all, the relative small sample size (*n* = 217) and the involvement of a single center limited the generalization of the results. We are looking to match our data with other carotid plaque biobanks but have so far failed to identify any with tissues available for protein and mRNA analysis. In addition, as previously acknowledged, we were not able to identify the precise mechanism by which CRP is deposited within the plaque.

In conclusion, CRP was stained at a protein level within carotid plaques despite almost undetectable mRNA levels. Intraplaque CRP directly correlates with parameters of vulnerability (such as M1 macrophage subset). High CRP levels (>2.9%) in the upstream portions of atherosclerotic plaques can predict MACEs at 18-month follow-up. On the contrary, serum hs-CRP levels were neither associated with intraplaque parameters of vulnerability nor associated with MACEs. Our results, even if preliminary, indicate that intraplaque rather than systemic CRP might be a proatherosclerotic factor potentially useful to better assess the CV risk in patients suffering from severe carotid atherosclerosis. Further studies are required to validate intraplaque CRP as a reliable marker of subclinical atherosclerotic disease.

## Figures and Tables

**Table 1:** Clinical characteristics of the overall cohort at admission.

	Overall cohort (*n* = 217)
*Demographics*	
Age, yr. (IQR)	72 (67.0–77.5)
Males, number (%)	136 (62.7)
Systolic BP^*∗*^, mmHg (IQR)	135 (130–143.75)
Diastolic BP, mmHg (IQR)	80 (80–90)
Waist circumference, cm (IQR)	90 (87–97)
Carotid stenosis, % (IQR)	80 (70.0–85.0)
Hypertension, number (%)	157 (72.4)
Active smokers, number (%)	54 (24.9)
Previous smokers, number (%)	93 (42.9)
Type 2 diabetes, number (%)	42 (19.4)
Dyslipidaemia, number (%)	125 (57.6)
Chronic CAD^†^, number (%)	41 (18.9)

*Medications*	
RAAS^‡^ inhibitors, number (%)	109 (50.2)
ACE-I^§^, number (%)	13 (6.0)
ARBs^‖^, number (%)	96 (44.2)
*β*-Blockers, number (%)	59 (27.2)
Calcium antagonists, number (%)	67 (30.9)
Diuretics, number (%)	28 (12.9)
Statins, number (%)	114 (52.5)
Antiplatelet drugs, number (%)	221 (82.2)
Aspirin, number (%)	129 (59.4)
Thienopyridine, number (%)	51 (23.5)
Anticoagulants (heparin), number (%)	11 (5.1)
Oral antidiabetics, number (%)	28 (12.9)
Insulin, number (%)	8 (3.7)

Data are expressed as median (interquartile range [IQR]) or number (percentages [%]).

^*∗*^BP: blood pressure.

^†^CAD: coronary artery disease.

^‡^RAAS: renin-angiotensin-aldosterone system.

^§^ACE-I: angiotensin converting enzyme inhibitor.

^‖^ARBs: angiotensin receptor blockers.

**Table 2:** Laboratory findings of the overall cohort at admission.

	Overall cohort (*n* = 217)
*Hematology*	
Total WBC^*∗*^, number × 10^9^/L (IQR)	7.10 (6.18–8.30)
Neutrophils, number × 10^9^/L (IQR)	4.54 (3.55–5.46)
Lymphocytes, number × 10^9^/L (IQR)	1.77 (1.42–2.15)
Monocytes, number × 10^9^/L (IQR)	0.44 (0.35–0.55)
Platelets, number × 10^9^/L (IQR)	229 (189.50–276.50)
Red blood cells, number × 10^12^/L (IQR)	4.70 (4.40–4.95)

*Chemistry*	
Serum total-c^†^, mg/dL (IQR)	194 (165–224.25)
Serum LDL-c^‡^, mg/dL (IQR)	114.90 (88.20–142.75)
Serum HDL-c^§^, mg/dL (IQR)	49 (41.25–61.75)
Serum TAG^‖^, mg/dL (IQR)	118 (90–162.25)
Fibrinogen, mg/dL (IQR)	3.69 (3.15–4.24)
Fasting glycemia, mg/dL (IQR)	101 (91–115.75)
hs-CRP^#^, *μ*g/mL (IQR)	2.38 (0.90–4.95)
IL-6^*∗∗*^, pg/mL (IQR)	2.16 (0.68–3.83)

Data are expressed as median (interquartile range [IQR]) or number (percentages [%]).

^*∗*^WBC: white blood cells.

^†^Total-c: total cholesterol.

^‡^LDL: low-density lipoprotein.

^§^HDL: high-density lipoprotein.

^‖^TAG: triglycerides.

^#^hs-CRP: high sensitivity c-reactive protein.

^*∗∗*^IL: interleukin.

**Table 3:** Distribution of inflammatory biomarkers within upstream and downstream portions of carotid plaques.

	Upstream	Downstream	*p* value
Neutrophil, cells/mm^2^	2.36 (0.93–5.93)	4.33 (1.20–10.68)	**0.002**
Total collagen, %	29.29 (18.28–38.57)	16.52 (8.39–22.11)	**<0.001**
Collagen I, %	10.29 (5.37–15.20)	5.30 (2.39–8.23)	**<0.001**
Collagen III, %	12.14 (8.20–18.23)	5.28 (2.93–8.98)	**<0.001**
CRP^*∗*^, %	1.54 (0.61–2.78)	1.04 (0.44–2.18)	0.070
Smooth muscle cells, %	4.53 (3.01–9.00)	2.78 (1.53–4.27)	**<0.001**
Total macrophages (CD68^+^)^†^, %	5.80 (2.72–10.23)	6.47 (2.24–13.94)	0.205
M^‡^1 subset (CD86^+^), %	1.33 (0.49–3.06)	3.21 (1.24–7.11)	**<0.001**
M1 subset (HLA-DR^+^)^§^, %	10.47 (6.07–18.30)	9.50 (5.81–14.30)	0.124
M2 subset (CD163^+^), %	1.66 (0.56–3.83)	1.37 (0.22–3.30)	**0.049**

Data are expressed as median (interquartile range [IQR]).

^*∗*^CRP: c-reactive protein.

^†^CD: cluster of differentiation.

^‡^M: macrophage.

^§^HLA-DR: human leukocyte antigen-antigen D related.

**Table 4:** Correlation between intraplaque CRP and inflammatory biomarkers in upstream and downstream plaques.

Upstream			Downstream		
CRP^*∗*^, % vs.	*r*	*p* value	CRP^*∗*^, % vs.	*r*	*p* value
Neutrophils/mm^2^	0.208	**0.008**	Neutrophils/mm^2^	0.087	0.275
Total collagen, %	0.063	0.430	Total collagen, %	−0.035	0.659
Collagen I, %	−0.008	0.918	Collagen I, %	−0.046	0.560
Collagen III, %	−0.138	0.082	Collagen III, %	−0.030	0.710
Smooth muscle cells, %	−0.315	**<0.001**	Smooth muscle cells, %	0.028	0.726
Total macrophages (CD68^+^)^†^, %	0.158	**0.046**	Total macrophages (CD68^+^)^†^, %	0.097	0.222
M^‡^1 subset (CD86^+^), %	0.113	0.151	M^‡^1 subset (CD86^+^), %	0.193	**0.014**
M1 subset (HLA-DR^+^)^§^, %	0.161	**0.042**	M1 subset (HLA-DR^+^)^§^, %	0.226	**0.004**
M2 subset (CD163^+^), %	0.031	0.694	M2 subset (CD163^+^), %	0.344	**<0.001**

Correlations were performed by Spearman's rank correlation coefficient.

^*∗*^CRP: high sensitivity c-reactive protein.

^†^CD: cluster of differentiation.

^‡^M: macrophage.

^§^HLA-DR: human leukocyte antigen-antigen D related.

**Table 5:** Relationship between serum hs-CRP and intraplaque parameters.

Upstream			Downstream		
hs-CRP^*∗*^, *μ*g/mL vs.	*r*	*p* value	hs-CRP^*∗*^, *μ*g/mL vs.	*r*	*p* value
Neutrophils/mm^2^	0.045	0.547	Neutrophils/mm^2^	0.107	0.147
Total collagen, %	−0.108	0.150	Total collagen, %	−0.017	0.819
Collagen I, %	−0.118	0.116	Collagen I, %	0.103	0.167
Collagen III, %	−0.038	0.614	Collagen III, %	0.064	0.393
CRP, %	−0.088	0.268	CRP, %	−0.013	0.871
Smooth muscle cells, %	−0.053	0.482	Smooth muscle cells, %	−0.031	0.673
Total macrophages (CD68^+^)^†^, %	0.016	0.832	Total macrophages (CD68^+^)^†^, %	0.056	0.451
M^‡^1 subset (CD86^+^), %	0.038	0.630	M^‡^1 subset (CD86^+^), %	−0.101	0.207
M1 subset (HLA-DR^+^)^§^, %	−0.098	0.222	M1 subset (HLA-DR^+^)^§^, %	−0.034	0.671
M2 subset (CD163^+^), %	0.055	0.481	M2 subset (CD163^+^), %	−0.055	0.485

Correlations were performed by Spearman's rank correlation coefficient.

^*∗*^CRP: high sensitivity c-reactive protein.

^†^CD: cluster of differentiation.

^‡^M: macrophage.

^§^HLA-DR: human leukocyte antigen-antigen D related.

**Table 6:** Cox proportional hazards model showing the predictive value of upstream CRP expression (cutoff > 2.9%) toward MACE occurrence during 18 months of follow-up.

	Univariate model			Multivariate model		
	HR^*∗*^	95% CI^†^	*p* value	HR	95% CI	*p* value
MACEs^‡^						
Upstream CRP^§^	6.22	1.49–26.05	**0.012**	8.57	1.89–38.77	**0.005**
Age	0.96	0.90–1.03	0.286	0.93	0.84–1.02	0.142
Gender, male	1.01	0.30–3.46	0.984	0.63	0.144–2.81	0.550
hs-CRP^‖^	0.78	0.57–1.06	0.114	0.49	0.23–1.01	0.053
Statin use	1.65	0.48–5.64	0.423	1.41	0.32–6.22	0.648

^*∗*^HR: hazards ratio.

^†^CI: confidence interval.

^‡^MACEs: major adverse cardiovascular events.

^§^CRP: c-reactive protein.

^‖^hs-CRP: high sensitivity c-reactive protein.
